# COVID-19: an ongoing public health crisis in the Philippines

**DOI:** 10.1016/j.lanwpc.2021.100160

**Published:** 2021-04-27

The Philippines is contending with one of the worst COVID-19 outbreaks in southeast Asia. As of April 18, 2021, there were 926 052 cases of SARS-CoV-2 infection and 15 810 deaths recorded. WHO has warned that the country's health-care system risks being overwhelmed. From March 29, 2021, a new round of lockdown was implemented in Manila and four surrounding provinces to suppress the new surge of infections. Although lockdown measures help control the spread of the virus, they only offer a short-term solution.

The pandemic has heavily hit the country in multiple ways. As an archipelagic country made up of more than 7000 islands, the Philippines is among the most vulnerable countries in the world to natural disasters. In addition, the longstanding battle with infectious diseases has been compounded with the rise in non-communicable diseases due to lifestyle changes and an increase in risk behaviours. These issues have predisposed the population to severe negative effects of the COVID-19 pandemic. The economy shrank almost 10% in 2020, which pushed more people into poverty. Besides the direct health losses due to the pandemic and the associated policy response, there are indirect health losses that are hard to estimate--for example, when health-care resources were reprioritised away from other important areas.

Case isolation, contact tracing, and physical distancing are recognised as the backbone of effective COVID-19 control. A prerequisite of successful implementation of these strategies is to have a robust public health system and sufficient workforce, which was inadequate and insufficient in the country even before the pandemic. The limited investment in health-care infrastructure and a shortage of health-care workers curtail the system, while inequalities in health-care delivery further jeopardise access to services. According to *The Philippines Health System Review* published by WHO in 2018, there were 23 beds per 10 000 individuals in the National Capital Region, and this number is less than ten per 10 000 individuals for the rest of the country. Public and privately owned health systems are supposed to be complementary in health-care service delivery, but no effective measure exists to regulate the expanding private sector, leading to a high amount of out-of-pocket expenses for health care; for example, more than half of total health spending was out-of-pocket in 2018. The COVID-19 pandemic puts further pressure on the fragmented public health system. Along with this fragmentation, the insufficient response from the government has resulted in a delay in contact tracing and mass testing, an overwhelmed medical system, and slow vaccine roll-out.

Parallel with public health policies, mass vaccination is another viable solution to this pandemic. There are 600 000 doses of the CoronaVac vaccine (Sinovac Life Sciences) donated from China and more than 525 600 doses of the ChAdOx1 nCoV-19 vaccine (Oxford--AstraZeneca) from the COVAX scheme arriving in the country. The shortage of COVID-19 vaccines and the slow vaccine roll-out has been criticised by the public; however, vaccine hesitancy is another public health issue that needs addressing. Public confidence in vaccines has dropped markedly since the Dengvaxia controversy and is likely to affect the willingness of people to accept COVID-19 vaccines. Concerns over data transparency, rare side-effects, and government accountability could further push people away from vaccination.

In February, 2019, the Philippines passed the Universal Health Care (UHC) law to ensure equitable access to quality and affordable health-care services for the entire population. The implementation of UHC can minimise the existing discrepancy in health-care systems. There is an urgent need for the country to gain control of the pandemic through mass testing, better contact tracing, and planning beyond vaccine acquisition and roll-out. These positive actions will not only help the country to recover from the pandemic, but also support UHC as a long-term goal to strengthen pandemic preparedness and response capability in the future.

*The Lancet Regional Health – Western Pacific*

